# *Coccidioides posadasii *infection alters the expression of pulmonary surfactant proteins (SP)-A and SP-D

**DOI:** 10.1186/1465-9921-5-28

**Published:** 2004-12-10

**Authors:** Shanjana Awasthi, D Mitchell Magee, Jacqueline J Coalson

**Affiliations:** 1 Department of Pathology, University of Texas Health Science Center at San Antonio, San Antonio, TX, USA; 2 Department of Microbiology and Immunology, University of Texas Health Science Center at San Antonio, San Antonio, TX, USA; 3 Center for Biomedical Inventions, University of Texas Southwestern Medical Center, Dallas, TX, USA

**Keywords:** Surfactant proteins, *Coccidioides posadasii*

## Abstract

**Background:**

Coccidioidomycosis or Valley Fever is caused by *Coccidioides *in Southwest US and Central America. Primary pulmonary infection is initiated by inhalation of air-borne arthroconidia. Since, lung is the first organ that encounters arthroconidia, different components of the pulmonary innate immune system may be involved in the regulation of host defense. Pulmonary surfactant proteins (SP)-A and SP-D have been recognized to play an important role in binding and phagocytosis of various microorganisms, but their roles in *Coccidioides *infection are not known.

**Methods:**

In this study, we studied the changes in amounts of pulmonary SP-A, SP-D and phospholipid in murine model of *Coccidioides posadasii *infection, and binding of SP-A and SP-D to Coccidioidal antigens. Mice were challenged intranasally with a lethal dose of *C. posadasii *(n = 30 arthroconidia) and bronchoalveolar lavage fluid (BALF) samples were collected on day 10, post infection. In another group of animals, mice were immunized with protective formalin killed spherule (FKS) vaccine prior to infection. The concentrations of BALF SP-A, SP-D, total phospholipid were measured using enzyme linked immunosorbent assay and biochemical assays.

**Results:**

We found that in lavage fluid samples of *C. posadasii *infected mice, the concentrations of total phospholipid, SP-A and SP-D were 17 % (SEM 3.5, p < 0.001), 38 % (SEM 5.8, p < 0.001) and 4 % (SEM 1.3, p < 0.001) of those in lavage fluid samples of non-infected control mice, respectively. However, the concentrations of SP-A and SP-D remained unchanged in BALF samples of *C. posadasii *protected mice after immunization with FKS vaccine. Also, we found that both SP-A and SP-D bind to Coccidiodal antigens.

**Conclusion:**

Our results suggest that the *C. posadasii *infection perturbs the pulmonary SP-A, SP-D, and phospholipids, potentially enabling the disease progression and promoting fungal dissemination.

## Background

Coccidioidomycosis or Valley Fever is a fungal disease caused by the biphasic, highly virulent, soil-fungus *Coccidioides immitis *or *posadasii *[1]. It is endemic in the southwest regions of US, Northern Mexico and parts of Central America [2]. *C. posadasii or C. immitis*, are the most virulent fungal pathogens enlisted in Select Agent list and pose a risk for bioterrorism [3]. The primary infection is acquired by inhalation of air-borne, mycelial phase arthroconidium that converts into endosporulating spherule in the lung. Clinical manifestations of the disease range from pulmonary infection to a more severe fatal mycosis involving extra-pulmonary tissues in 1–10% of the infected people [1-4]. Previous studies suggest that Th1 cell mediated immunity protects individuals against *Coccidioides *[5,6]. However, information is lacking regarding the pulmonary innate immune components that may play a critical role in regulation of immune responses against *Coccidioides*.

At alveolar level in the lung, the innate immune system is composed of many cell types and chemical mediators, including surfactant. The pulmonary surfactant is a complex mixture of lipids (88–90%) and proteins (10–12%), synthesized by type II epithelial cells and Clara cells. It lines the alveoli, and helps in maintaining normal lung function [7]. Among four different surfactant proteins, surfactant proteins-A (SP-A) and D (SP-D) are members of the "Collectin" family [8]. In the past, several studies have suggested that both SP-A and SP-D play an important role in innate host defense against various viral, fungal and bacterial pathogens [9,10]. More evidence for the pulmonary collectins' role in host defense comes from studies on SP-A- deficient mice that are susceptible to intra-tracheal Group B *Streptococci *[11], *Pseudomonas aeruginosa *[12], and Respiratory Syncytial Virus [13]. Also, intranasally administered SP-D has been found to reduce replication of Respiratory Syncytial Virus in the lungs of infected mice [14]. Both SP-A and SP-D, have been classified as secretory pattern-recognition receptors that can bind to a variety of pathogens and help in clearance [9,15]. Recent evidences indicate that in addition to their pathogen recognition property, SP-A and SP-D also play an important role in stimulating immuno-regulatory pathways [15]. However, the collectins' role in coccidioidomycosis is not known.

This study focuses on analyzing the changes in amounts of the SP-A and SP-D in the bronchoalveolar lavage fluid (BALF) samples from mice infected with lethal dose of *C. posadasii *and *C. posadasii *protected mice after immunization with protective formalin killed spherule (FKS) vaccine, and binding of pulmonary collectins to Coccidioidal antigens.

## Methods

### Mice

BALB/c and C57BL6 mice (6 weeks old female) from Jackson Laboratory (Bar Harbour, ME) were used in this study. Both mouse strains are susceptible to *C. posadasii *infection. The BALB/c mice were used to study the changes in pulmonary surfactant after intranasal challenge with *C. posadasii*. And, the C57BL6 mice were used to study the changes in pulmonary surfactant after vaccination with protective FKS vaccine. Mice were housed in Biosafety Level-3 animal facility at UTHSCSA and provided with food and water ad libitum. All experimental animal care and treatment protocols were reviewed and approved by Institutional Animal Care and Use Committee.

#### Coccidioides posadasii

*C. immitis *(now *posadasii*) Silveira strain, cultured on 1 % glucose-0.5 % yeast extract agar (GYE), was used for infecting the mice [1]. The arthroconidia were harvested in endotoxin-free 0.15 M saline (Baxter Health Care Products, Deerfield, IL) from 6–8 weeks old mycelial phase cultures grown on GYE plates. The arthroconidia suspension was passed over a sterile cotton column to remove hyphal elements and arthroconidia were enumerated by hemacytometer counts. The viable cfu counts were confirmed pre- and post-infection by plate cultures on GYE agar. All the experiments with *C. posadasii *were carried out in Biosafety Level-3 facility at UTHSCSA.

### Intranasal Challenge with *C. posadasii *Arthroconidia

Mice were anaesthetized after intramuscular injection of ketamine- xylazine (75 μg/g body weight ketamine and 10 μg/g body weight xylazine) and were then challenged intranasally with a lethal dose of arthroconidia (n = 30, fresh harvest of *C. posadasii *arthroconidia) suspended in endotoxin-free 0.15 M NaCl (Baxter Health Care Corp, Deerfield, IL) using sterile pyrogen-free microtip. Mice were held in an upright position for 1–2 min to resume normal breathing after injection. Control mice were challenged with equal volume of endotoxin-free 0.15 M NaCl.

### Preparation of *Coccidioide*-FKS vaccine and Immunization of Mice

*C. posadasii *(strain *Silveira*)arthroconidia were used to prepare FKS as described earlier [16]. Briefly, arthroconidia were inoculated in modified Converse medium containing Tamol and cultured while shaking at 180 rpm at 40°C in 20 % CO_2 _incubator. The spherules were collected from the harvested culture, washed in endotoxin-free water and killed with 1 % formalin. FKS preparation was checked for sterility and lyophilized. C57BL6 female mice (age 6 weeks old) were immunized intramuscularly twice and subcutaneously once at one week interval with FKS (0.7 mg/dose each time). The mice in FKS immunized, infected group were then challenged intranasally with 30 *C. posadasii *arthroconidia, 15 days after last immunization, as described above.

### Fungal Burden Assay

Mice were anaesthetized as mentioned above, prior to sacrifice on day 10, post intranasal infection. This standard procedure was used for intranasal injection since it does not cause respiratory depression during anaesthesia. The lung and spleen tissues were collected in sterile 0.15 M NaCl for studying fungal load.

The fungal burden was studied by plating ten fold dilutions of lung and spleen homogenates in 0.15 M saline on Mycosel agar plates (BD Biosciences, Franklin Lakes, NJ) and incubating for 72 h at 30°C. The cfu counts were recorded and normalized with organ weight.

### Collection and Processing of BALF

At the time of necropsy, we collected BALF by injecting 1 ml endotoxin-free 0.15 M NaCl solution (Baxter Health Care Corp, Deerfield, IL) three times, via an angiocatheter (BD Biosciences, San Diego, CA) placed in the trachea. The volume of the input solution was kept constant (3 ml total) and approximately, 90–95 % of the solution was recovered consistently. The BALF was centrifuged at 500 rpm for 10 min at 4°C to remove cells. The cell free BALF supernatant was filtered through 0.2 μm syringe filters (Nalge Nunc International, Rochester, NY) and stored at -80°C for further analysis.

### Total Protein and Lipid Analysis

The total protein concentration was measured in BALF specimens using micro bicinchonic acid protein assay kit (Pierce, Rockford, IL) against bovine serum albumin (BSA) standard protein. The total phospholipid content in lipid extracts of BALF specimens was determined using the method of Stewart, against Dipalmitoyl-phosphatidylcholine (DPPC, Avanti Polar Lipids, Alabaster, AL) standard solutions [17,18]. Briefly, the lipid extract of BALF specimens and DPPC standard solutions was completely dried under compressed nitrogen gas. The dried lipids were dissolved in chloroform and mixed with 1 ml of 2.7% ferric chloride and 3% ammonium thiocyanate in glass tubes. The mixture was vortex mixed for 1 min and centrifuged at 200 rpm for 5 min. The bottom red lower layer of phospholipids and ammonium ferro-thiocyanate complex was collected and absorbance was read at 488 nm.

### Western Blotting

The BALF and lung tissue homogenate samples (total protein 10–50 μg) were run on 10% sodium dodecyl sulfate- polyacrylamide gel electrophoresis (SDS-PAGE) running gel and transferred on nitrocellulose membranes (Schleicher & Schuell, Keene, NH) overnight at 15 mA current. The nonspecific sites on the membrane with transferred proteins were blocked by 15% nonfat milk in Tris-buffered saline containing 0.05% tween 20 (TBST). The membrane was washed and incubated for 1 h with diluted (1:500) primary anti-human SP-A polyclonal antibody raised in rabbit (obtained from Dr. Richard J. King, UTHSCSA, San Antonio, TX) or anti-mouse SP-D antibody (kindly provided by Dr. Jo Rae Wright, Duke University Medical Center, Durham, NC). After washing the membrane with TBST, the membrane was incubated for 1 h with 1:10,000 diluted alkaline phosphatase conjugated anti-rabbit IgG raised in goat (Sigma Chemical Co, St. Louis, MO). The immunoreactive bands were observed by alkaline phosphatase conjugate system (Biorad, Hercules, CA). Purified human SP-A (kindly provided by Dr. Richard J. King, UTHSCSA, San Antonio, TX) and recombinant human SP-D (kindly provided by Dr. Erika C. Crouch, Washington University in St. Louis, St. Louis, MO) were run with the samples.

The Coccidioidal antigens: lysates and filtrates of Coccidioidin (CDN), prepared as a toluene-induced lysate of young *C. posadasii *mycelia (obtained from Dr. Rebecca A. Cox, UTHSCSA, San Antonio, TX, [19]) were also run to check the cross-reactivities of anti-SP-A and SP-D antibodies to fungal antigens.

### Enzyme-Linked Immunosorbent Assay (ELISA) for SP-A and SP-D

**Figure 1:**
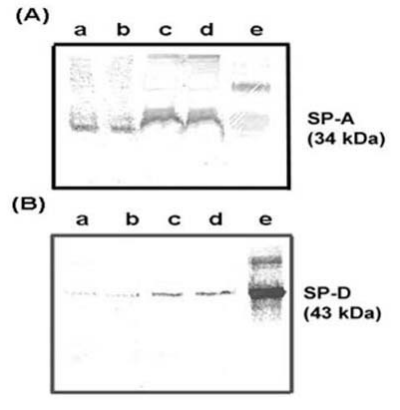
Western blot for (A) SP-A and (B) SP-D proteins in mouse lung. Lanes (a, b): 2.5 μg total lavage fluid protein (c, d): 100 μg of total lung tissue homogenate protein from two healthy, non-infected BALB/c mice, and (e): 10 ng purified human SP-A or recombinant SP-D protein.

The concentrations of SP-A and SP-D were measured in BALF samples as described earlier [20]. The antibodies against SP-A and SP-D reacted with 34 kDa (SP-A) and 43 kDa (SP-D) immunroreactive bands in BALF and lung tissue homogenates (Fig 1). For measuring the lavage concentrations of SP-A and SP-D, the indirect ELISA procedure was used [20]. Briefly, the wells of Immulon 4 strips (Dynatech, Chantilly, VA) were coated overnight with purified human SP-A or recombinant human SP-D antigens (standards) and diluted BALF (three different dilutions) in 0.1 M NaHCO_3_, pH 9.6. The wells were washed three times with deionized water, and nonspecific sites were blocked with a buffer containing 0.25% BSA, 0.05% tween 20, 0.17 M boric acid and 0.12 M NaCl, pH 8.5. The wells were washed and incubated for 2 h with rabbit anti-human SP-A or rabbit anti-mouse SP-D antibody. After washing the wells, the horseradish peroxidase conjugated anti-rabbit IgG antibody (Sigma, St. Louis, MO) was added. After incubation for 2 h, the wells were washed again and incubated with tetramethylbenzidine substrate reagent (Sigma Chemical Co. St. Louis, MO). The reaction was stopped by adding 50 μl of 2 N H_2_SO_4 _and read at 450 nm spectrophotometrically. The regression coefficient for a least-square linear fit to the standard curve of SP-A and SP-D was 0.99. The limits of detection for SP-A and SP-D were 2 ng/ml.

### Binding of SP-A and SP-D to Coccidioidal Antigens

A microtiter well based method [21] was used to study the SP-A and SP-D interactions with Coccidioidal antigens (CDN-lysate and CDN-filtrate). Briefly, microtiter wells (Immulon 4; Dynatech, Chantilly, VA) were coated with 50 μl of CDN-lysate (10 μg/ml diluted in 0.1 M NaHCO_3 _buffer, pH 9.6) or CDN-filtrate (10 μg/ml diluted in 0.1 M NaHCO_3 _buffer, pH 9.6) or BSA (10 μg/ml diluted in 0.1 M NaHCO_3 _buffer, pH 9.6) at room temperature. The nonspecific binding was blocked with phosphate buffered saline (pH 7.4) containing 0.1% triton-X 100 and 3% nonfat milk (buffer A). The purified human SP-A and recombinant human SP-D diluted in 20 mM Tris (pH 7.4) containing 0.15 M NaCl, 5 mM CaCl_2 _and 1 mg/ml BSA were then added to the wells and incubated for 3 h at 37°C. The wells were then washed with buffer A and incubated for 1 h at room temperature with diluted (1:1000 in buffer A) anti-SP-A and anti-SP-D antibodies. After washing the wells, the horseradish peroxidase conjugated anti-rabbit IgG antibody (Sigma, St. Louis, MO) was added. After incubation for 2 h, the wells were washed again and incubated with tetramethylbenzidine substrate reagent (Sigma Chemical Co. St. Louis, MO). The reaction was stopped by adding 2 N H_2_SO_4 _and read at 405 nm spectrophotometrically.

The coating of Coccidioidal antigens (CDN-lysate and CDN-filtrate) to the plates was confirmed using a positive control antibody that recognizes Coccidioidal antigens as described earlier [22]. The alkaline phosphatase-conjugated rat anti-mouse IgG antibody (Zymed, San Francisco, CA) served as secondary detection antibody.

### Statistics

Statistical analyses of the data (t-test or ANOVA) were done using Prism Software (Graphpad Software, San Diego, CA). The p value <0.05 was considered significant.

## Results

### Pathological status

All of the *C. posadasii *infected mice survived till the day of sacrifice (day 10 post infection). However, the mice were lethargic and lost body weight (Table 1). Abscess like lesions were quite evident on gross examination of the lung. The total wet lung weights were increased in *C. posadasii *infected mice.

**Table 1:** Body weights (g) of *C. posadasii *infected and non-infected BALB/c mice (n = 10 of each type). Values are shown as Mean (SEM) of one representative experiment of two independent experiments.

**Days post challenge → Mice ↓**	**Day 0**	**Day 10**
Non-infected	18.36 (0.29)	19.46 (0.23) **
*C. posadasii *infected	17.77 (0.39)	16.35 (0.53) *, #

** p < 0.01 as compared to non-infected control mice at day 0.

*p < 0.05, # p < 0.0001 as compared to *C. posadasii *infected mice at day 0 and non-infected mice at day 10, respectively (t-test).

The mean protein content of BALF samples from infected mice was 788 μg versus 326 μg protein in BALF samples from non-infected saline injected control mice, after 10 days of intranasal infection (p < 0.05, Table 2). In contrast, the phospholipid concentration was reduced in BALF samples from *C. posadasii *infected mice (58 μg) when compared to non-infected saline injected controls (165 μg, p < 0.05).

**Table 2:** Total protein and phospholipid contents in BALF samples from non-infected and *C. posadasii *infected BALB/c mice (n = 5 of each type). Values are shown as Mean (SEM) from one represenative experiment of two independent experiments.

**Mice**	**Total protein (μg)**	**Total phospholipid (μg)**
Non-infected	326.0 (26.9)	165.5 (10.8)
*C. posadasii *infected	788.7 (248.6)*	58.7 (15.7)*

* p < 0.05 as compared to non-infected control mice (t-test).

### The amounts of SP-A, SP-D and phospholipid are reduced in BALF samples from *C. posadasii *infected mice

**Figure 2:**
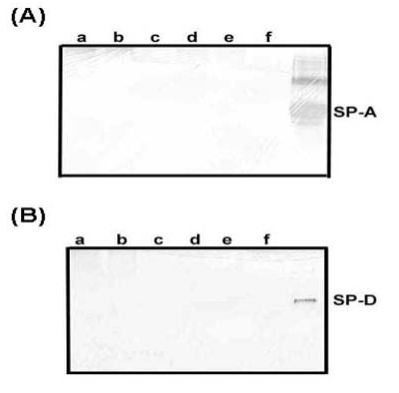
Western blot of CDN-lysate and CDN-filtrate for crossreactivity with (A) anti-human SP-A and (b) anti-mouse SP-D antibodies. Lanes (a): 20 μg, (b): 10 μg and (c): 1 μg CDN-filtrate protein. Lanes (d): 20 μg, (e): 10 μg and (f): 1 μg CDN-lysate protein and last lane: 10 ng purified human SP-A protein or recombinant SP-D protein.

**Figure 3:**
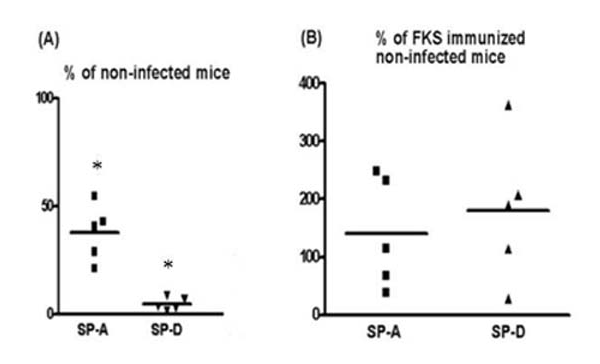
SP-A and SP-D levels in BALF samples from (A) *C. posadasii *infected BALB/c mice (ng/μg protein, % of non-infected control mice, n = 5 of each type) (B) FKS immunized, *C. posadasii *infected C57BL6 mice (protected mice) (ng/μg protein, % of FKS immunized non-infected mice, n = 5 of each type). The data are shown from one representative experiment of two independent experiments. * p < 0.001 (ANOVA)

The anti-human-SP-A and anti-mouse SP-D antibodies recognized 34 kDa and 43 kDa monomer bands of SP-A and SP-D in BALF and lung tissue samples (Fig 1). The upper bands of approximately 68 kDa and 85 kDa size (dimer of SP-A and SP-D protein) were also visible in lanes e of Figure 1A and 1B due to incomplete reduction, respectively. There were no differences in the detectable isoforms of SP-A or SP-D in the BALF samples from infected mice as compared to non-infected control mice (data not shown). The antibodies did not cross-react with Coccidioidal antigens in CDN lysate or CDN filtrate (Fig 2). The amounts of SP-A and SP-D were significantly reduced in BALF samples from *C. posadasii *infected BALB/c mice when compared to saline injected, non-infected control mice after 10 days of intranasal challenge (p < 0.001) (Fig 3A). No significant changes were observed in the amounts of SP-A and SP-D in BALF samples collected from BALB/c mice, 5 days after intranasal challenge with *C. posadasii *(data not shown).

**Figure 4:**
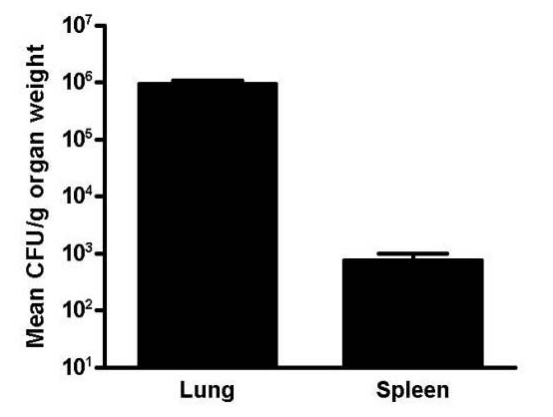
Fungal load in the lung and spleen tissues of *C. posadasii *infected BALB/c mice (n = 5). The numbers of fungal colonies (CFU) were normalized with organ weight (g).

The fungal colonies of *C. posadasii *were recovered from both lung and spleens of infected animals indicating the presence of active infection (Fig 4). Recovery of fungus in the spleen provides evidence of dissemination of *C. posadasii to *extra-pulmonary organs.

### Lavage SP-A and SP-D levels are unaltered in *C. posadasii *protected mice

No significant changes were seen in SP-A or SP-D levels in BALF samples of protected (FKS immunized, *C. posadasii *arthroconidia infected) C57BL6 mice when compared to FKS immunized, non-infected mice (Fig 3B). Also, there was no significant change in total protein content in BALF samples of FKS immunized, *C. posadasii *arthroconidia infected mice (890.9 μg) versus FKS immunized, non-infected mice (538.4 μg).

### SP-A and SP-D bind to Coccidioidal antigens

**Figure 5:**
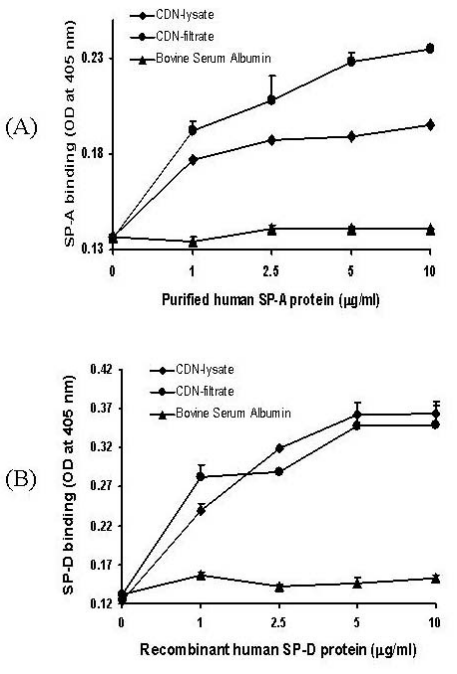
Binding of (A) SP-A and (B) SP-D to Coccidioidal antigens (CDN-lysate and CDN-filtrate). The binding of 1–10 μg/ml purified human SP-A or recombinant SP-D proteins was detected in CDN-lysate or CDN-filtrate or BSA coated wells (0.5 μg/well). Results are from one representative experiment of two independent experiments performed in duplicate. Values are shown as mean+SEM. In some cases, the error bars are smaller than the symbols.

We further examined the binding of SP-A and SP-D to Coccidioidal antigens (CDN-lysate and CDN-filtrate) coated onto microtiter wells (Fig 5). Both SP-A and SP-D bound to coccidioidal antigens, but not to BSA in a concentration-dependent manner (Fig 5). Binding of SP-A to CDN-lysate and CDN-filtrate antigens was saturable, and maximum SP-A binding was reached between 2.5–5 μg/ml and 5–10 μg/ml, respectively (Fig 5A). Similarly binding of SP-D to CDN-lysate and CDN-filtrate antigens was also saturable, and maximum SP-D binding was reached between 5–10 μg/ml (Fig 5B).

## Discussion

In the present study we found that the levels of pulmonary surfactant collectins were altered in the lungs of *C. posadasii *infected mice, but were intact in lungs of *C. posadasii *protected mice after immunization with protective FKS vaccine. Furthermore, our results suggest that both SP-A and SP-D bind to Coccidioidal antigens. This is the first study where the amounts of SP-A and SP-D were measured in BALF samples from mice infected with lethal dose of *C. posadasii *and the binding of pulmonary collectins to Coccidioidal antigens was assessed. Since lung is the first organ of the body that comes into contact with air-borne *C. posadasii *arthroconidia, we hypothesized that the pulmonary surfactant may play an important role in regulating the immune response against *C. posadasii*. Among four surfactant proteins, SP-A and SP-D, interact with most of the clinically important fungal pathogens including *Pneumocystis carinii *[23]*Cryptococcus neoformans *[24], *Aspergillus fumigatus *[25] and *Candida albicans *[26]. SP-D has been shown to bind to *C. albicans *and directly inhibit growth by aggregation of the organism without involvement of macrophage dependent phagocytosis [27]. On the other hand, surfactant proteins also induce phagocytosis, activation and killing of *A. fumigatus *conidia and *C. neoformans *by alveolar macrophages and neutrophils [28,29]. In support of these findings, further evidence comes from a study by Madan et al., that suggests that the introduction of recombinant SP-D improves the lung function and increases the survival rate of mice infected with *A. fumigatus *[25]. The decrease in amounts of SP-A and SP-D during *C. posadasii *infection versus the unaltered amounts in the lungs of protected mice and binding of collectins to Coccidioidal antigens indicate that pulmonary collectins may be involved in uptake/phagocytosis of *C. posadasii *by antigen presenting cells and downstream immune regulation.

Besides changes in amounts of SP-A and SP-D, a decrease in the amount of BALF phospholipids was also observed in *C. posadasii *infected mice (Table 2). Earlier, Sheehan et al., [29] and Hoffman et al., [30] have reported similar findings of reduced surfactant phospholipid level in BALF samples of rats and humans infected with *Pneumocystis carinii *[29,30]. To date, however, the information is lacking concerning how surfactant phospholipids may be involved in host defense [31]. Likewise, the mechanisms underlying the decrease of BALF surfactant in murine model of Coccidioidomycosis remain to be defined. We speculate that the reduction in the collectins and phospholipids could be either due to metabolic dysfunction of pulmonary type II epithelial cells during *C. posadasii *infection or due to their utilization in the binding and uptake of *C. posadasii *by local antigen presenting cells. The metabolic pathways of pulmonary type II epithelial cells may be affected by secondary inflammatory mediators, such as TNF-α or IL-1β, secreted by inflammatory cells during *C. posadasii *infection. A variety of host inflammatory mediators and substances such as, cytokines (TNF-α) and growth factors are released during infection and inflammation. These cytokines and growth factors affect the synthesis and secretion of pulmonary surfactant by pulmonary epithelial cells [32,33]. In the present study we found slightly increased levels of SP-D in *C. posadasii *protected mice, but not of SP-A (Fig 2). We speculate that the difference could be due to diverse mechanisms for regulation of SP-A and SP-D expression. Probably the cytokines and chemokines that are released as a result of FKS vaccination (protective immune response) increase the SP-D expression, but do not affect the SP-A expression. As reported earlier, the expression of SP-A and SP-D is differentially regulated during lung infection [34]. In future, more studies are warranted to understand the mechanism of the alterations in levels of surfactant phospholipids, SP-A and SP-D.

At present, the treatment of *C. posadasii *infected patients with disseminated disease is not very effective [35]. Treatment with anti-fungal agents is most often related to relapse of the infection and side effects on the body. Earlier, the FKS based immunization has been found protective against *Coccidioides *infection in murine model of coccidioidomycosis, but failed in humans [35]. More pre-clinical studies on developing different vaccine strategies are underway. Since *C. posadasii *and *C. immitis *are highly virulent organisms, cause endemic infection, and pose a risk for bioterrorism, there is an urgent need for discovery of improved therapeutic drugs and regimens or preventive vaccines [3,35].

## Conclusions

In future, clearance experiments after *in vivo *administration of artificial or natural surfactant in *C. posadasii *infected mice may be useful in determining their therapeutic usefulness. We speculate that the findings from our present study would initiate similar studies to understand the role of pulmonary innate immune components in infectious diseases caused by *C. posadasii *or other virulent respiratory pathogens.

## Authors' Contributions

SA designed and co-ordinated the study, performed assays and statistical analysis and drafted the manuscript. JJC assessed lung pathology. DMM prepared and immunized mice with FKS. All authors read and approved the final manuscript.
